# Cerebral Cavernous Malformation Proteins in Barrier Maintenance and Regulation

**DOI:** 10.3390/ijms21020675

**Published:** 2020-01-20

**Authors:** Shu Wei, Ye Li, Sean P. Polster, Christopher R. Weber, Issam A. Awad, Le Shen

**Affiliations:** 1 Graduate Program in Public Health and Preventive Medicine, Wuhan University of Science and Technology, Wuhan 430081, China; shuwza@gmail.com; 2 Department of Pathology, The University of Chicago, Chicago, IL 60615, USA; yli170@bsd.uchicago.edu (Y.L.); cweber@bsd.uchicago.edu (C.R.W.); 3 Section of Neurosurgery, Department of Surgery, The University of Chicago, Chicago, IL 60615, USA; spolster@uchicago.edu (S.P.P.); iawad@uchicago.edu (I.A.A.)

**Keywords:** cerebral cavernous malformation, endothelial barrier, epithelial barrier, Rho, ROCK, MEKK3

## Abstract

Cerebral cavernous malformation (CCM) is a disease characterized by mulberry shaped clusters of dilated microvessels, primarily in the central nervous system. Such lesions can cause seizures, headaches, and stroke from brain bleeding. Loss-of-function germline and somatic mutations of a group of genes, called *CCM* genes, have been attributed to disease pathogenesis. In this review, we discuss the impact of *CCM* gene encoded proteins on cellular signaling, barrier function of endothelium and epithelium, and their contribution to CCM and potentially other diseases.

## 1. Introduction

One of the key functions of endothelial and epithelial cells is to create a barrier that separates different tissue compartments, and in the case of skin, epithelial cells separate body and outer environment. Compromised barrier function leads to abnormal mixing of different tissue components, which can contribute to pathogenesis of many diseases. In this review, we focus on a group of proteins that participates in the development of a neurovascular disease, cerebral cavernous malformation (CCM), and examine their impact on cellular signaling and barrier function.

## 2. Clinical Features of CCM

**Figure 1:**
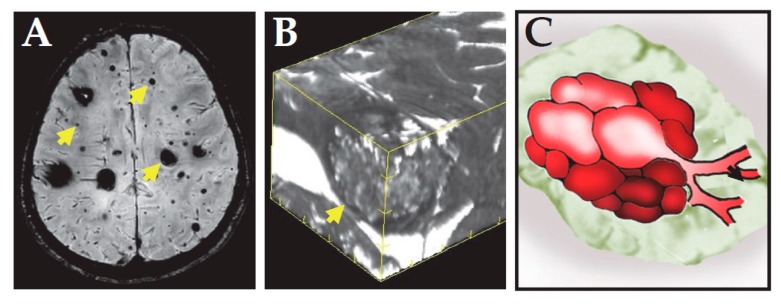
Radiological presentation of CCM. (**A**) MRI image of the brain of a familial CCM patient. Susceptibility weighted imaging showed multiple dark CCM lesions with various sizes. Arrows indicate representative lesions. (**B**) 3D reconstruction of T2 weighted imaging of a CCM lesion. It shows the lesion is not uniform, but with popcorn appearance. The arrow indicates the location of the lesion. (**C**) Schematic presentation of a CCM lesion showing it is composed of nested dilated microvessels.

**Figure 2:**
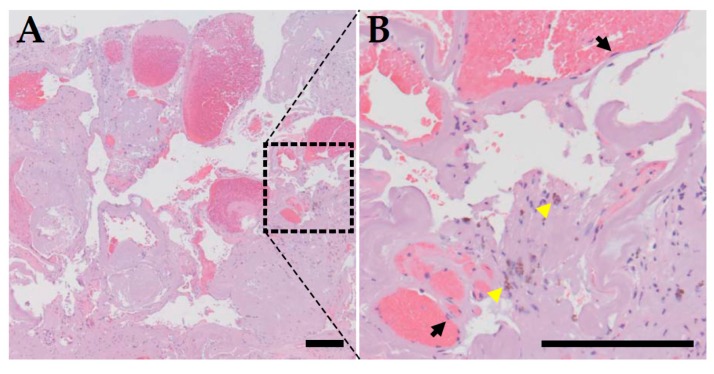
Histopathological presentation of CCM. (**A**) H&E staining of a surgically resected CCM lesion. It is composed of clusters of thin walled dilated microvessels with no supporting smooth muscle cells beneath the endothelial cell layer and no intervening brain parenchyma. Thrombi are present within the lumen of capillaries within the CCM lesion. (**B**) High power image of the boxed region of panel A. Black arrows point to individual endothelial cells lining the inner surface of dilated capillaries, and yellow arrowheads point to hemosiderin deposition adjacent to the capillaries, a sign of chronic bleeding. Bar = 200 μm.

CCM (also known as cavernous angioma) disease is characterized by the development of abnormally dilated capillaries, primarily in the central nervous system (Figure 1) [1]. Grossly, these lesions appear to be blood filled, mulberry shaped clusters of thin-walled small vessels. Histologically, the nested microvessels have little supporting tissue and intervening parenchyma, and the dilated vessels are lined by a single layer of dysmorphic endothelial cells. Thrombi frequently form in these vessels, and hemosiderin deposits can be seen adjacent to these capillaries, indicating chronic bleeding (Figure 2). CCM patients are mostly diagnosed by magnetic resonance imaging initiated due to neurological changes, including headache, seizures, and other neurological deficits, such as nausea or vomiting, weakness or numbness, slurred speech, and altered vision. About 25% to 50% of CCM patients do not have clinical symptoms, and only a small fraction of these patients is identified incidentally [2,3]. The prevalence of CCM is about 0.5% in the general population [4,5], and about 70% to 80% of CCM patients have one lesion, and the other 20% to 30% of CCM patients have more than one lesion [6,7]. Most of the patients with one lesion have the sporadic form of the disease without a family history, while the majority of the patients who have more than one lesion have a family history with autosomal dominant Mendelian inheritance.

## 3. Genetics of CCM

Based on linkage analyses, three gene loci (*CCM1* [7q21-22], *CCM2* [7p15-p13], and *CCM3* [3q25.2-q27]) have been identified in the germ-line of familial cases [8,9,10]. Subsequently, the genes within these loci are identified to be *CCM1/KRIT1*, *CCM2/MGC4607*, and *CCM3/PDCD10* [11,12,13,14,15,16]. Of all familial CCM patients, ~60% have *CCM1* mutations, ~20% have *CCM2* mutations, ~10% have *CCM3* mutations, and a minority of familial CCM patients do not have mutations in these three genes [17]. Although mutations of *KRIT1*, *CCM2*, and *PDCD10* genes are all associated with histologically identical CCM lesions, patients with *PDCD10* mutations have the most severe phenotype, with earlier symptomatic onset [18,19]. A large fraction of mutations identified in patients are located in the coding region of *CCM* genes and are loss-of-function mutations [20]. DNA sequencing of lesional tissue and endothelial cells from familial CCM patients showed that in addition to germ-line mutations, these harbor somatic mutations of *CCM* genes, suggesting a two-hit mechanism for CCM pathogenesis [21,22]. Somatic mutations in the same *CCM* genes have been identified in sporadic lesions, indicating that loss of CCM function also contributes to sporadic disease development [23]. This also suggests that biomarkers and therapeutic targets aimed at the familial disease will also apply in sporadic CCM cases.

CCM proteins are conserved molecules. Orthologs of all three *CCM* genes have been identified in *Caenorhabditis elegans*. The *KRIT1* ortholog *kri-1* germline affects animal longevity and germ cell survival [24,25], and *ccm-2* participates in such processes [26]. The *PDCD10* ortholog *ccm-3* is required for excretory canal organization and germline tube development through affecting a large array of cellular events, including actomyosin organization, cell polarity and endocytic recycling [26,27,28]. In zebrafish, *krit1* and *ccm2* loss leads to dilation of major vessels, with spreading of endothelial cells [29], and a C-terminally truncated PDCD10 causes a similar phenotype [30]. Although *Ccm* heterozygous knockout mice have little or no potential to develop CCM–like lesions in the brain, when they are on a genetically instable background (*Msh2*^−/−^ or *Trp53*^−/−^), these mice have a significantly higher lesion burden [19,31,32]. These findings demonstrate that loss of heterozygosity is likely an important driving force for CCM pathogenesis. Mouse studies further show that KRIT1, CCM2, and PDCD10 participate in CCM pathogenesis. Deletion of *Krit1*, *Ccm2*, and *Pdcd10* genes all cause embryonic lethality due to cardiovascular defects [33,34,35]. Conventional homozygous *Krit1* and *Ccm2* deletion both cause defects in branchial arch artery formation [33,34], while *Pdcd10* deletion causes vasculogenesis and hematopoiesis defects [35]. When embryonic lethality is circumvented by tamoxifen-induced postnatal deletion of floxed *Ccm* genes, CCM-like lesion formation ensues, primarily in the cerebellum, suggesting they are CCM disease causing genes [36,37,38,39]. Consistent with human studies, mice with *Pdcd10* deletion also showed a more severe phenotype than mice with *Krit1* or *Ccm2* mutations, indicating PDCD10 may affect KRIT1 and CCM2-independent events [19]. Recent evidence reveals that clonally expanded mutated endothelial cells only comprise a fraction of cells lining CCM lesions, suggesting endothelial cells with *CCM* deletions may co-opt endothelial cells without *CCM* mutations to participate in CCM disease [40].

## 4. CCM Proteins and Their Interactions

**Figure 3:**
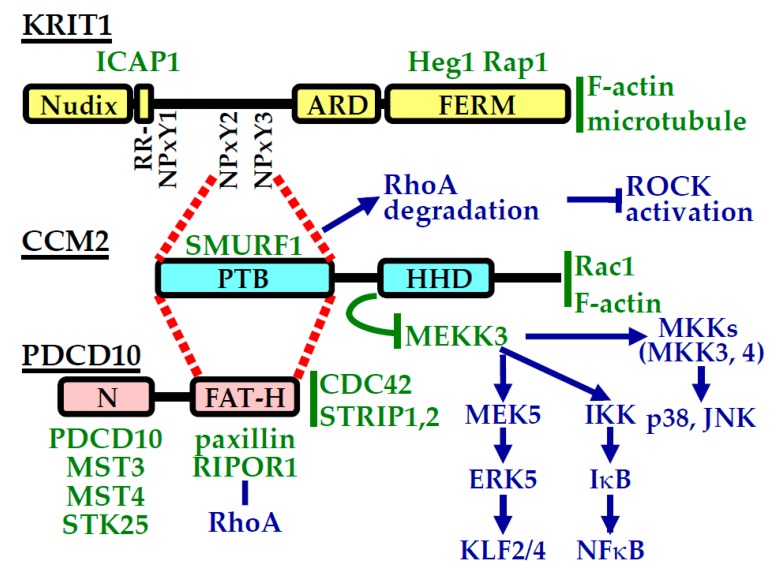
CCM protein domain organization and protein interactions. CCM protein domain organizations are presented schematically. Direct interaction partners are shown in green letters. Locations of the letters indicate rough interaction sites for these binding proteins. If a binding site is unknown, the binding partner is listed to the right of each CCM protein. Key pathways affected by CCM protein and their interaction partners are shown in blue letters. Dashed red lines indicate interaction sites between individual CCM proteins.

KRIT1 (Krev interaction trapped protein-1, CCM1) is the largest of the three CCM proteins, with 529 amino acid residues [41]. It was first identified through its binding to the small GTPase Rap1 (also called Krev-1), and it is comprised of an N-terminal Nudix domain, three NPxY/F motifs, an ankyrin-repeat region, and a C-terminal FERM (band 4.1, ezrin, radixin, moesin) domain (Figure 3). Through its N-terminal Nudix domain and NPxY/F motif containing region, KRIT1 interacts with the β1-integrin binding protein ICAP1 to limit β1-integrin activation [42,43]. The KRIT1 FERM domain binds to a transmembrane protein Heg1 and the small GTPase Rap1 and is important for KRIT1 to localize to the plasma membrane [41,44,45,46]. Consistent with its role in cytoskeletal regulation, KRIT1 also directly associates with microtubules [47].

CCM2 is a 444 amino acid residue protein, with a phosphotyrosine-binding domain (PTB) at its N-terminus and a C-terminal harmonin-homology domain (HHD) [13,48]. It was first characterized as an osmosensing scaffolding protein that binds to small GTPase Rac1 and protein kinases MEKK3 and MKK3 [49]. CCM2 is central to the CCM protein complex organization, as it can bind to both KRIT1 and PDCD10 (programmed cell death 10, CCM3) (Figure 3) [50,51,52]. The CCM2 PTB domain binds directly with the KRIT1 NPxY/F motif, and LD-like motif of CCM2 (within the linker region between the PTB and HHD domains) binds to the focal adhesion targeting (FAT) homology domain of PDCD10 [51,52,53,54]. Binding between KRIT1 and CCM2 is important for CCM2 localization [51,54], while the interaction between CCM2 and PDCD10 controls CCM2 and PDCD10 protein stability, as CCM2 depletion decreases cellular PDCD10 protein content, and PDCD10 depletion reduces CCM2 protein abundance [53]. CCM2 also associates with F-actin, bringing the actin regulating small GTPase Rac1 to the proximity of the actin cytoskeleton [55]. A paralog of CCM2, CCM2L, also exists [56]. Although CCM2L can bind to KRIT1 and compete with CCM2 for KRIT1 binding, it does not bind to PDCD10 [56]. Similar to CCM2, CCM2L also interacts with MEKK3 [57], but the significance of CCM2L for CCM disease pathogenesis and its effect on CCM protein complex organization and function remains poorly defined [58].

PDCD10 (CCM3) protein has 212 amino acid residues and consists of an N-terminal dimerization domain and a C-terminus FAT homology domain (Figure 3) [59]. It was first discovered as a gene upregulated during myeloid cell apoptosis [60]. In addition to binding to CCM2 [50], PDCD10 can bind to components of another protein complex, the striatin interacting phosphatase and kinase (STRIPAK) complex, through its dimerization domain. These proteins include striatin itself and germinal center kinase (GCK) III group of serine/threonine protein kinases MST4/MASK, MST3/STK24, and STK25/YSK1/SOK1 and other STRIPAK complex components, including STRIP1/FAM40A and STRIP2/FAM40B [61,62,63,64,65,66,67]. Although PDCD10 can bind to CCM2, PDCD10 primarily resides within the STRIPAK complex, rather than the CCM protein complex, in cells [63,64]. Furthermore, PDCD10 can bind to an array of other proteins, including paxillin, PTPN13, UNC13D [50,67,68,69,70]. Similar to KRIT1 and CCM2, PDCD10 also interacts with cytoskeletal regulating small GTPases. Cdc42 can co-immunoprecipitate with PDCD10, and Cdc42 deletion causes a CCM-like phenotype, suggesting Cdc42 and PDCD10 resides in the same CCM pathogenic pathway [71]. In addition, PDCD10 can directly bind to RIPOR1/FAM65A, a RhoA associated protein, providing a link between PDCD10 and RhoA signaling [72].

## 5. CCM Proteins and Cellular Signaling

Because each of the CCM proteins has a multitude of interaction partners, it is not surprising that these proteins can impact multiple signaling pathways and cellular processes, including endothelial to mesenchymal transition, autophagy, exocytosis, and Golgi complex organization [63,69,72,73,74,75]. One of the best understood CCM controlled signaling pathways is the RhoA-Rho-associated coiled-coil kinase (ROCK) signaling. Decreased expression of any of the CCM proteins leads to increased RhoA and ROCK activity [19,30,34,54,76,77], which in turn increases myosin regulatory light chain (MLC) phosphorylation, causing actomyosin contraction that affects cell migration and intercellular junction integrity. Through its PTB domain, CCM2 can bind to the E3 ubiquitin ligase Smad ubiquitin regulatory factor 1 (Smurf1) [76,78], which ubiquitinates RhoA to promote its degradation [78]. In the absence of CCM2, Smurf1-mediated RhoA degradation is reduced, leading to RhoA accumulation and increased ROCK activity [78]. Depletion of PDCD10 and its binding partners STK25, STRIP1/FAM40A, STRIP2/FAM40B, and RIPTOR/FAM65A all increase MLC phosphorylation, indicating PDCD10 may affect RhoA-ROCK activity through these proteins [19,30,65]. The enhanced RhoA-ROCK signaling is a critical component of CCM pathogenesis, which is further detailed below.

Another relatively well understood CCM-regulated pathway is the MEKK3 signaling. As discussed above, CCM2 directly interacts with MEKK3 [49]. Both *Krit1* and *Ccm2* deletion leads to activation of the MEKK3-MEK5-ERK5-KLF2/4 signaling cascade, causing increased Adamts4/5 expression [57,79,80]. These changes disrupt both embryonic cardiac development and promote CCM-like lesion formation in neonatal mice [79,80]. Consistent with the findings that CCM2 negatively regulates MEKK3, and MEKK3 is required for immune related receptor signaling [81,82,83,84], MEKK3 activating ligands lipopolysaccharide (LPS), IL-1β, and pI:pC can all promote CCM-like lesion formation [85]. There is some evidence that aberrantly activated MEKK3 signaling can lead to increased RhoA-ROCK signaling, but the exact mechanism for this potential crosstalk and its contribution to CCM disease need to be further elucidated [79,80,85,86].

CCM proteins have also been implicated in cell death regulation. The *C. elegans KRIT1* ortholog *kri-1* is required to promote irradiation-induced germ cell death through a cell-nonautonomous fashion [25], while in neuroblastoma cells, CCM2 is critical for the TrkA receptor tyrosine kinase to induce tumor cell death [87,88]. As its name suggests, PDCD10 has also been associated with apoptosis regulation. In endothelial cells, overexpression of PDCD10 promotes endothelial apoptosis, and in cardiomyocytes, PDCD10 expression is required for ischemic reperfusion injury-induced cell death [89,90]. However, the exact effect of PDCD10 on apoptosis is still under debate. For example, PDCD10 is up-regulated during oxidative stress, but one report suggested such upregulation promotes tumor cell survival, while another report suggested such upregulation enhances apoptosis [91,92]. Thus, how CCM proteins affect cell death and proliferation to impact human health and disease remains to be further explored.

## 6. CCM Proteins Participate in Endothelial Barrier Maintenance and Regulation

Early morphological studies showed that CCM lesions are lined by altered endothelial cells with disrupted cell–cell connections, including tight junctions [93,94]. Using MRI based in vivo permeability measurements, it is now clear that CCM lesions have increased vascular permeability [95,96]. In white matter regions away from CCM lesions, patients with familial CCM disease (harboring a germline mutation) have higher baseline permeability than patients with sporadic disease, indicating *CCM* mutations globally affect blood–brain barrier function [95,96]. Furthermore, baseline brain white matter vascular permeability can be used to distinguish familial CCM patients with non-aggressive and aggressive disease, and between stable and non-stable CCM disease [95,96]. These data suggest blood–brain barrier defect regulates CCM clinical disease presentation.

Consistent with patient-based studies, cell culture and mouse studies demonstrated how CCM proteins may affect endothelial barrier function. All three CCM proteins can limit RhoA-ROCK signaling in endothelial cells, although PDCD10 may use a mechanism distinctive of KRIT1 and CCM2 [19,54,65]. The small GTPase RhoA and the other two CCM protein binding small GTPases Rac1 and Cdc42 are cytoskeletal regulators that control barrier function [97,98,99]. In the case of RhoA, its effector ROCK can either directly or indirectly induce MLC phosphorylation, leading to perijunctional actomyosin contraction, which in turn causes intercellular junction remodeling to increase paracellular permeability [100]. Indeed, decreased KRIT1, CCM2 and PDCD10 expression all promote MLC phosphorylation, stress fiber formation, junctional protein redistribution, and barrier dysfunction in endothelial cells [19,34,54,101].

In addition to maintaining baseline endothelial barrier, KRIT1 also participates in endothelial barrier regulation. While tumor necrosis factor (TNF) increased arteriole and venule permeability in wild type mice, it failed to induce barrier loss in *Krit1* heterozygous knockout mice [101,102]. In contrast, histamine-induced vascular permeability increase occurred normally in *Krit1* heterozygous knockout mice [101,102]. However, another report suggests KRIT1 is required for preservation of endothelial barrier following stimuli [103]. KRIT1 depletion in cultured endothelial cells attenuated prostacyclin-induced perijunctional F-actin accumulation and tightening of endothelial barrier and enhanced cyclic stretch-induced Rho activation and endothelial barrier disruption [103]. In vivo studies further showed that *Krit1* heterozygous knockout exacerbated barrier loss induced by combined treatment of high tidal volume mechanical ventilation and TRAP6, a thrombin receptor activating peptide. This treatment also increased protein and cell content of bronchoalveolar lavage fluid, indicating partial KRIT1 loss participates in lung damage [103]. These data suggest KRIT1 may participate in endothelial barrier regulation in a stimulus-dependent manner and contribute to endothelial dysfunction-related diseases.

Because of the robust ROCK activation in CCM depleted endothelial cells, ROCK became a leading target for novel CCM therapy. ROCK inhibition not only reverses CCM depletion-induced stress fiber formation and barrier loss in vitro but also limits *Ccm* deletion-induced loss of endothelial barrier function in vivo [19,34,54,101]. Pharmacological studies further show that ROCK inhibition by fasudil, atorvastatin, and a newly identified ROCK2 specific inhibitor limits CCM-like lesion formation in multiple mouse models of CCM [104,105,106], highlighting ROCK inhibition may be a valid therapy for CCM disease. This proof of concept is currently being tested in a clinical trial (NCT02603328) [107].

Besides RhoA-ROCK signaling, additional cellular processes have been implicated for CCM proteins to regulate endothelial barrier. Vascular endothelial growth factor (VEGF) not only promotes endothelial growth, but also increases endothelial permeability [108]. It has been demonstrated that loss of KRIT1 and PDCD10, but not CCM2, increases VEGF production in endothelial cells, and VEGF in turn acts on VEGFR2 to increase endothelial permeability [109]. However, existing evidence also suggests that PDCD10 is required for proper VEGFR2 signaling [35], indicating the relationship between CCM proteins and VEGF and its signaling may be complex. In KRIT1 depleted cells or heterozygous knockout mice, endothelial reactive oxygen species (ROS) production is elevated, at least partially, through upregulated NAPDH oxidase expression [102,110]. When an endothelial targeting ROS scavenger was used, the increased vascular permeability was reduced in KRIT1 deficient mice, demonstrating ROS production also plays a role for KRIT1 to regulate endothelial barrier [102]. However, the molecular mechanisms for ROS to affect barrier function in endothelium, in the presence or absence of KRIT1, remain to be elucidated.

## 7. Tight Junctions and CCM Disease

One of the major determinants of the endothelial barrier is the tight junction. In contrast to well demarcated tight junction, adherens junction, and gap junction domains within the apical junctional complex of epithelial cells, these domains are frequently mixed at cell–cell contact sites between endothelial cells [111]. Such junctions can vary significantly in endothelial cells of different origins. Microvascular endothelial cell bodies can have a thickness of 0.3 μm, with cell–cell junction depth of ~0.5–0.9 μm, while endothelial cells from arteries and high endothelial venules the cell–cell contact sites may reach 3–10 μm in height [111]. In the brain, the endothelial cells, pericytes at the abluminal side of endothelial cells, and astrocyte end feet together form the neurovascular unit to create the highly impermeable blood–brain barrier [112]. At the endothelial junctional complex, the adherens junction component VE-cadherin provides adhesive force at the cell–cell junctions, and the tight junction proteins are critical for limiting permeability between individual endothelial cells.

The tight junction seals the paracellular space between individual cavity lining cells and is created and maintained by a large number of transmembrane proteins. The four-transmembrane-domain-containing claudin family consists of more than 25 members in mammals. Some of the claudins (including claudin-1, -3, -5) are barrier forming, while some claudin family members are forming size and charge selective pores that allow charged ions and small molecules to pass (including claudin-2, -10, -15) [113]. In the brain microvascular endothelium, the most dominantly expressed claudin is the barrier forming claudin-5 [114,115]. Although claudin-5 is not required for brain microvascular endothelial tight junction organization, its knockout increased brain microvascular permeability, leading to neonatal death [116]. The four-transmembrane domain-containing tight-junction-associated MARVEL protein (TAMP) family contains occludin, tricellulin, and marveld3 [117], and these proteins generally impact macromolecular permeability [118,119]. Occludin knockout itself does not disrupt normal epithelial tight junction organization, but causes brain calcification, particularly around small vessels [120]. Patients with homozygous recessive occludin mutations have a more severe brain phenotype, with band-like calcification with simplified gyration and polymicrogyria [121]. This suggests occludin plays a critical role in brain development, likely through affecting brain endothelial function. Additional tight junction proteins belong to the immunoglobulin superfamily of proteins with a single transmembrane domain (e.g., junctional adhesion molecule A, JAM-A and Coxsackie and adenovirus receptor, CAR) and popeye family of proteins with three transmembrane domains (Popdc1/Bves). In the intestine, JAM-A maintains proper epithelial macromolecular barrier function and limits intestinal inflammation [122,123], and endothelial JAM-A promotes leukocyte transmigration [73,124]. Similarly, CAR participates in epithelial barrier maintenance [125], and CAR affects shear stress induced endothelial immune response [126].

Multiple plaque proteins concentrate at the cytoplasmic side of the tight junction. These proteins typically bind to multiple transmembrane tight junction proteins, other tight junction plaque proteins, and the cytoskeleton, thus stabilize tight junction organization. Zonula occludens (ZO) family proteins (ZO-1, -2, -3) is a well-studied family of tight junction plaque proteins [127]. They can bind to almost all transmembrane tight junction proteins, heterodimerize among different ZO proteins, and associate with the actin cytoskeleton [127]. ZO-1 knockout mice are embryonic lethal, with defects in vascular endothelial cells [128], a finding supported by in vitro endothelial cell studies [129]. Cingulin family is another group of tight junction plaque proteins (cingulin, paracingulin/cingulin-like/JACOP) that can interact with occludin, JAM-A, ZO proteins, myosin and actin filaments, which are also required for proper endothelial function, including brain endothelial barrier function [130,131].

Many CCM affected signaling events can regulate the tight junction. As discussed above, Rho-ROCK signaling increases MLC phosphorylation to impact actomyosin contraction, which in turn regulates tight junction protein expression and localization [132,133,134,135]. In addition, ROCK can directly phosphorylate occludin and claudin-5, and such phosphorylation events are associated with blood brain barrier dysfunction [136]. Interaction between endothelial cells and basement membrane induces β1-integrin engagement, increases MLC phosphorylation in an MLC kinase and ROCK -dependent fashion to promote claudin-5, occludin, and ZO-1 reorganization at the cell–cell junction [137]. This pathway is likely affected by CCM proteins through KRIT1 binding to the β1-integrin signaling inhibitor ICAP-1, a protein that can also bind to ROCK [138,139,140]. The KRIT1 binding small GTPase Rap1 enhances tight junction protein localization at endothelial cell–cell contact sites and promotes endothelial barrier function [141]. Consistent with this, the Rap1 activating guanine-nucleotide-exchange factor EPAC also maintains endothelial barrier, prevents VEGF and TNF-induced endothelial permeability increase, and limits claudin-5, occludin, and ZO-1 disorganization at the cell–cell junctions [142]. Another small GTPase, Rasip1, is an effector of Rap1, which down-regulates RhoA activity through ArhGAP29 [143,144,145]. Rasip1 can also interact with the KRIT1 interacting transmembrane protein Heg1 [146], thus KRIT1 can bring Rasip1 and Rap1 close to one another through KRIT1-Heg1 interaction. Furthermore, engagement between individual JAM-A molecules at intercellular junctions can activate Rap1 to preserve epithelial and endothelial barrier functions through JAM-A interaction with the tight junction protein ZO-2, the adherens junction protein AF-6, and PDZ-GEF1/2 [147,148]. These data provide a complex signaling network for the tight junction proteins (JAM-A and ZO-2) and other cell surface adhesion molecules (β1-integrin and Heg1) to affect CCM-dependent cellular signaling pathways to impact tight junction barrier.

Consistent with such findings, resected CCM lesions have reduced occludin, claudin-5, and ZO-1 staining, and decreased tight junction protein expression has between associated with the tendency for local bleeding and edema [149,150]. In *Krit1* deleted brain microvascular endothelial cells, loss of claudin-5 and ZO-1 protein can be readily observed by immunofluorescent staining and western blot [151], and PDCD10 depletion in brain microvascular endothelial cells decreases claudin-5, occludin, and ZO-1 protein abundance, likely through an ERK1/2 and cortactin-dependent process [152]. A recent study suggests PDCD10 depletion in brain endothelial cells upregulates gap junction protein connexin 43 expression and increases gap junction communication, a phenomenon only minimally seen in KRIT1 or CCM2 depleted cells [153]. Such changes are associated with redistribution of tight junction proteins to gap junctions, and the connexin 43 gap junction inhibitor GAP27 can reverse tight junction disorganization and decrease endothelial barrier permeability in PDCD10 depleted cells [153]. These indicate increased gap junction function participates in tight junction disruption in *CCM3* disease. With such findings, it is likely that tight junction protein disorganization downstream of RhoA-ROCK signaling and gap junction is a key effector driving CCM pathogenesis, and it is possible that normalizing tight junction protein expression and localization at the cell–cell junctions can limit CCM development or lesional bleeding. However, the specific roles of tight junction proteins in CCM initiation and progression remain to be formally tested, likely by using transgenic or knockout mice.

## 8. CCM Proteins Impact Intestinal Homeostasis

In contrast to a plethora of studies on the function of CCM proteins in endothelial cells, we just start to appreciate their roles in epithelial cells. By investigating the effects of KRIT1 in β-catenin signaling, Glading and Ginsberg revealed KRIT1 depletion increases β-catenin transcriptional activity in both endothelial and epithelial cells [154]. This is functionally significant, as *Apc* mutation induced more intestinal polyp formation in *Krit1* heterozygous knockout mice with increased intestinal epithelial nuclear β-catenin accumulation [154]. A recent *C. elegans* study suggested KRIT1 can also form a complex with CCM2 to promote zinc transporter expression to cause Zn^2+^ storage in the intestinal granules, indicating KRIT1 may also impact intestinal epithelial transport [26].

**Figure 4:**
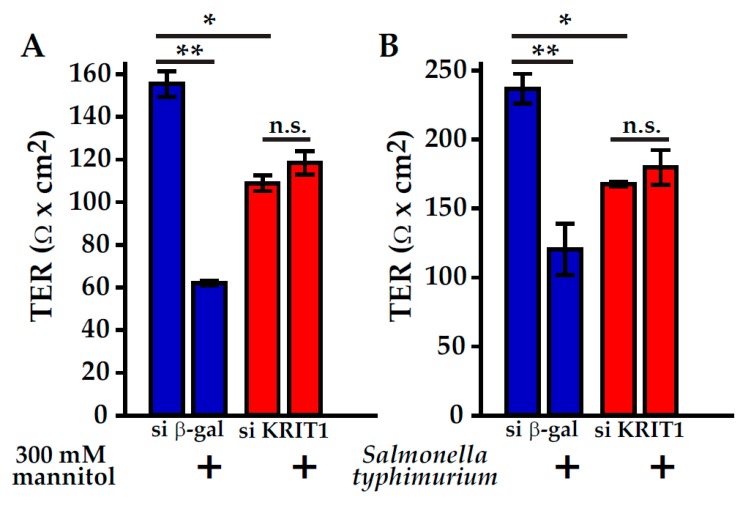
KRIT1 depletion limits pathophysiological stimuli-induced epithelial barrier dysfunction. KRIT1 depletion by stable transfection of a siRNA expressing plasmid decreased epithelial barrier function (A-B, assessed by transepithelial resistant (TER) measurements) in differentiated Caco-2 intestinal epithelial monolayers grown on semi-permeable Transwell inserts [155]. (**A**) Hyperosmotic stress induced by including 300 mM mannitol in Hank’s balanced salt solution caused barrier loss in control (siRNA against β-galactosidase transfected, blue bars) Caco-2 monolayers. In contrast, no barrier loss was induced in KRIT1 depleted (siRNA against KRIT1 transfected, red bars) Caco-2 monolayers. (**B**) *Salmonella typhimurium* (strain ATCC 14028) infection by including bacteria in apical culture media caused barrier loss in control, but not KRIT1 depleted Caco-2 monolayers. Mean with standard error (triplicate samples) are shown. One-way ANOVA analysis with Bonferroni correction was used (* *p* < 0.05, ** *p* < 0.01).

Despite these findings, it was not known if CCM proteins can regulate barrier function in epithelium. Our group addressed this question by studying KRIT1 function in intestinal epithelial Caco-2 cells, a well characterized model to study intestinal epithelial barrier maintenance and regulation [155]. In this model, KRIT1 depletion caused a reduction of epithelial barrier function, characterized by selectively increased relative permeability of small cations, including Na^+^, to the anion Cl^-^ [155]. Such a change is consistent with decreased expression of claudin-1, a tight junction protein that limits small ion permeability, in KRIT1-depleted Caco-2 cells [155,156]. In contrast to the effect of KRIT1 on endothelial cells, intestinal epithelial KRIT1 depletion does not induce MLC phosphorylation, and ROCK inhibition does not reverse KRIT1 depletion-induced barrier loss [155]. This indicates that KRIT1 regulates epithelial and endothelial barrier function through distinct mechanisms. In Caco-2 monolayers, decreasing actomyosin contractility by inhibiting either ROCK or myosin ATPase activity both reduced epithelial barrier function, along with elevated permeability to both small and large cations. These changes are inhibited in KRIT1-depleted Caco-2 monolayers, indicating KRIT1 also participates in actomyosin contraction-induced barrier regulation [155]. Furthermore, KRIT1-depleted epithelial monolayers are resistant to osmotic stress and enteric pathogen *Salmonella typhimurium*-induced epithelial barrier regulation (Figure 4), suggesting KRIT1 may impact gastrointestinal pathophysiology. With the above data, it is surprising to find that KRIT1 depletion exacerbates TNF-induced epithelial barrier loss. Mechanistic studies suggest this loss is due to aberrantly activated apoptosis in KRIT1-depleted monolayers, but we currently do not know how this occurs [155]. Nevertheless, these data suggest KRIT1 regulates epithelial barrier function through at least two distinct pathways: one is actomyosin and tight junction-dependent barrier maintenance and regulation, and the other is tight junction-independent epithelial apoptosis. Such findings not only point to a role for KRIT1 to mediate the crosstalk between distinctive epithelial barrier regulation pathways, but also suggest KRIT1 may coordinate tight junction barrier maintenance, regulation, and epithelial apoptosis to impact intestinal disease development.

An understanding of the potential contribution of the gastrointestinal tract to CCM disease development was stemmed from the surprising finding that neonatal mice with the same induced endothelial specific *Ccm* deletion can have drastically different CCM-like lesion burdens when they were raised in different animal facilities [85]. Fecal microbiome analysis showed that mice susceptible to *Ccm* deletion-induced lesion formation have a Gram-negative bacteria rich microbiome relative to resistant mice. Such a fecal microbiome provides the cell wall product LPS as the ligand to activate the endothelial TLR4-MEKK3-KLR2/4 signaling pathway to promote CCM development [85]. This view is further supported by the finding that germ-free mice and mice treated with antibiotics have lower lesion burden [85]. Because familial CCM patients have genetic mutations of *CCM* genes in all organs and cell types, this study also raised the possibility that CCM could function in the gastrointestinal tract to influence CCM disease development. Indeed, when *Pdcd10* was deleted in the intestinal epithelium, it promoted endothelial *Pdcd10* deletion-induced lesion formation [157]. In contrast, intestinal epithelial specific deletion of *Krit1* does not impact endothelial *Krit1* deletion-induced lesion formation [157]. This finding may at least partially explain why patients with *PDCD10* mutations have a more aggressive CCM disease than patients with *KRIT1* mutations. In addition to impacting CCM-like disease development, constitutive intestinal epithelial specific *Pdcd10* deletion alone shortened mouse life span, reduced intestinal mucus layer thickness, enlarged goblet cells, and caused intestinal inflammation [157]. These findings indicate PDCD10 is required for intestinal homeostasis and may impact intestinal disease development, which needs to be further investigated.

## 9. Conclusions and Future Directions

Through their many binding partners, CCM proteins impact many cellular events. The most prominent effect of CCM proteins on cellular signaling is their ability to limit RhoA-ROCK activity and MEKK3-MEK5-ERK5-KLF signaling, events that are important for endothelial function and CCM lesion formation. Despite such detailed understanding, we are just starting to grasp the full spectrum of CCM protein functions. Understanding how CCM proteins affect endothelial function through a variety of pathways to impact CCM disease and identifying therapies to preserve and promote normal CCM function in the brain remain top priorities for CCM research. With the finding that CCM proteins also function in intestinal epithelial cells, it becomes pressing to understand CCM protein functions in the gut, in the context of both CCM disease and other intestinal disorders. It also points to a need to understand CCM protein signaling in other cell types and organs. Such studies will not only advance our understanding of CCM protein biology, but also provide targets to modulate cellular functions to benefit human health.

## Abbreviations

CCM	cerebral cavernous malformation
EPAC	Rap1 guanine-nucleotide-exchange factor
FAT	focal adhesion targeting
FERM	band 4.1, ezrin, radixin, moesin
GCK	germinal center kinase
HHD	harmonin-homology domain
JAM	junctional adhesion molecule
KRIT1	Krev interaction trapped protein-1
LPS	lipopolysaccharide
MLC	myosin regulatory light chain
PDCD10	programmed cell death 10
PTB	phosphotyrosine-binding domain
ROCK	Rho-associated coiled-coil kinase
ROS	reactive oxygen species
Smurf1	Smad ubiquitin regulatory factor 1
STRIPAK	striatin interacting phosphatase and kinase
TER	transepithelial resistance
TNF	tumor necrosis factor
VEGF	vascular endothelial growth factor
ZO	zonula occludens
